# Meat Consumption as a Risk Factor for Type 2 Diabetes

**DOI:** 10.3390/nu6020897

**Published:** 2014-02-21

**Authors:** Neal Barnard, Susan Levin, Caroline Trapp

**Affiliations:** 1 Physicians Committee for Responsible Medicine, The George Washington University School of Medicine, 5100 Wisconsin Ave NW, Washington, DC 20016, USA; E-Mail: nbarnard@pcrm.org; 2 Nutrition Education, Physicians Committee for Responsible Medicine, 5100 Wisconsin Ave NW, Washington, DC 20016, USA; E-Mail: ctrapp@pcrm.org

**Keywords:** diabetes, meat, vegetarian, risk factor

## Abstract

Disease risk factors identified in epidemiological studies serve as important public health tools, helping clinicians identify individuals who may benefit from more aggressive screening or risk-modification procedures, allowing policymakers to prioritize intervention programs, and encouraging at-risk individuals to modify behavior and improve their health. These factors have been based primarily on evidence from cross-sectional and prospective studies, as most do not lend themselves to randomized trials. While some risk factors are not modifiable, eating habits are subject to change through both individual action and broader policy initiatives. Meat consumption has been frequently investigated as a variable associated with diabetes risk, but it has not yet been described as a diabetes risk factor. In this article, we evaluate the evidence supporting the use of meat consumption as a clinically useful risk factor for type 2 diabetes, based on studies evaluating the risks associated with meat consumption as a categorical dietary characteristic (*i.e.*, meat consumption *versus* no meat consumption), as a scalar variable (*i.e.*, gradations of meat consumption), or as part of a broader dietary pattern.

## 1. Introduction

Disease risk factors identified in epidemiological studies serve as important public health tools, helping clinicians identify individuals who may benefit from more aggressive screening or risk-modification procedures, allowing policymakers to prioritize intervention programs, and encouraging at-risk individuals to modify behavior and improve their health. According to the American Diabetes Association, the following factors justify testing for diabetes in asymptomatic adults of any age with a body mass index ≥25 kg/m^2^ [1].

(1) Physical inactivity
(2) First-degree relative with diabetes
(3) High-risk race/ethnicity (e.g., African American, Latino, Native American, Asian American, Pacific Islander)
(4) Women who delivered a baby weighing >9 lb or were diagnosed with gestational diabetes
(5) Hypertension (blood pressure ≥ 140/90 mmHg or on therapy for hypertension)
(6) High density lipoprotein cholesterol level < 35 mg/dL (0.90 mmol/L) and/or a triglyceride level > 250 mg/dL (2.82 mmol/L)
(7) Women with polycystic ovarian syndrome
(8) A1C ≥ 5.7%, impaired glucose tolerance, or impaired fasting glucose on previous testing
(9) Other clinical conditions associated with insulin resistance (e.g., severe obesity, acanthosis nigricans)
(10) History of cardiovascular disease

Risk factors have been based primarily on evidence from cross-sectional and prospective studies (e.g., the association between obesity and type 2 diabetes) demonstrated in the 1976–1980 National Health and Nutrition Examination Survey [2] and the association between ethnicity and diabetes described by the Centers for Disease Control and Prevention, based on several data sets [3]. Most risk factors do not lend themselves to randomized trials.

The utility of identifiable risk factors is illustrated by the link between excess body weight and diabetes risk. In a combined report of data from the Nurses’ Health Study and the Health Professionals Follow-up Study, men and women whose body mass indices were in the overweight range (25.0 kg/m^2^–29.9 kg/m^2^) were 4.6 and 3.5 times more likely to develop diabetes, respectively, compared with those whose body mass indices were below 25 kg/m^2^ [4]. In the Diabetes Prevention Program, individuals in the placebo or lifestyle treatment groups with body mass indices greater than 35 kg/m^2^ had roughly double the risk of developing diabetes during the 3.2-year follow-up period, compared with individuals with body mass indices below 30 kg/m^2^ [5]. As a result of these observations and similar findings in other studies, clinicians are encouraged to screen all overweight and obese adults for diabetes, rather than waiting to begin screening at age 45 [1]. In addition, federal policies, including the *Dietary Guidelines for Americans*, alert individuals regarding the risks of excess body weight [6].

While some risk factors are not modifiable, eating habits are subject to change through both individual action and broader policy initiatives. Although meat consumption is an easily ascertained and commonly investigated variable associated with diabetes risk [7,8,9,10,11,12,13,14,15,16,17], it has not yet been described as a diabetes risk factor. In this article, we evaluate the evidence supporting the use of meat consumption as a clinically useful risk factor for type 2 diabetes, based on studies evaluating the risks associated with meat consumption as a categorical dietary characteristic (*i.e.*, meat consumption *versus* no meat consumption), as a scalar variable (*i.e.*, gradations of meat consumption), or as part of a broader dietary pattern.

## 2. Findings

### 2.1. Risk Associated with Meat Consumption as a Categorical Variable

Researchers investigating relationships between diet and disease risk have sought to identify groups of individuals who differ on relevant dietary variables while remaining reasonably homogeneous in other respects. In this regard, Seventh-day Adventists have been an attractive population for study, because nearly all Adventists avoid tobacco, alcohol, and caffeine, while roughly half are omnivores and half are vegetarians, allowing researchers to identify the effects of dietary variations in an otherwise health-conscious population.

Three large Adventist cohorts have examined relationships between meat consumption and diabetes risk in both cross-sectional and prospective analyses (Table 1). The Adventist Mortality Study included a baseline survey of 24,673 white Seventh-day Adventists living in California in 1960, revealing 40% and 80% higher prevalences of diabetes among meat-consuming women (prevalence ratio = 1.4, 95% CI, 1.2–1.8) and men (prevalence ratio = 1.8, 95% CI, 1.3–2.5), respectively, compared with vegetarians, after adjustment for age and body weight [7]. Diabetes prevalence increased as the frequency of meat consumption increased.

During the 21-year follow-up of this cohort focusing on those who did not report diabetes at baseline, the mention of diabetes on a death certificate was used as a surrogate for diabetes prevalence [7]. Compared with those who avoided meat, the relative risk of having diabetes on a death certificate, adjusted for age, was 2.2 (RR = 2.2, 95% CI, 1.5–3.4) for meat-consuming men and 1.4 (RR = 1.4, 95% CI, 1.0–1.9) for meat-consuming women. Meat consumption was defined as having red meat or poultry at least once weekly (fish was reportedly rarely consumed in this cohort). Further adjustment for body weight weakened these associations; the increased risk remained significant for men, but not for women. Adjustment for body mass index may lead to an underestimate of risk, as is discussed further below.

The Adventist Health Study included a baseline survey of 34,192 non-Hispanic white California Adventists, showing that, age-adjusted, men and women who consumed meat had a 97% (OR = 1.97, 95% CI, 1.56–2.46, *P* = 0.0001) and 93% (OR = 1.93, 95% CI, 1.65–2.25, *P* = 0.0001) increased risk for diabetes, respectively, compared with those who avoided meat [8].

**Table 1:** Published studies of the relationship between meat consumption and risk of type 2 diabetes.

Meat as a Categorical Variable				
Study	Observation Period	Population	Findings	Adjustments
Adventist Mortality Study Snowdon *et al.* (1985) [7]	1960	24,673 white Seventh-day Adventists	Prevalence ratio and 95% CI for diabetes diagnosis: Men = 1.8 (1.3, 2.5); Women = 1.4 (1.2, 1.8)	Age and body weight
Adventist Mortality Study Snowdon *et al.* (1985) [7]	21-year follow-up	24,673 white Seventh-day Adventists	Relative risk for diabetes on death certificate: Men = 2.2 (1.5, 3.4); Women = 1.4 (1.0, 1.9)	Age
Adventist Health Study-1 Fraser (1999) [8]	1976	34,192 Seventh-day Adventists in California	Odds ratio and 95% CI for diabetes diagnosis: Men = 1.97 (1.56, 2.47, *p* = 0.0001); Women = 1.93 (1.65, 2.25, *p* = 0.0001)	Age
Adventist Mortality Study and Adventist Health Study-1 Tonstad *et al.* (2013) [11]	17-year follow-up	8401 Seventh-day Adventists	Odds ratio with 95% CI for diabetes diagnosis: 1.29 (1.08, 1.55)	Age and gender
Adventist Health Study-2 Tonstad *et al.* (2009) [10]	2002–2006	60,903 Seventh-day Adventists in North America	Odds ratio and 95% CI for diabetes diagnosis: 0.54 (0.49, 0.60)	Age, sex, ethnicity, education, income, physical activity, television watching, sleep habits, alcohol use, and body mass index
Adventist Health Study-2 Tonstad *et al.* (2013) [11]	2-year follow-up	41,387 Seventh-day Adventists	Odds ratio with 95% CI for diabetes diagnosis: 0.618 (0.0503, 0.760)	Age, body mass index, gender, ethnicity, income, and education
Meta-analysis Pan *et al.* (2011) [12]	4.6 to 28 years follow-up	442,101	Relative ratios and 95% CI for diabetes diagnosis + D1: 100 g unprocessed red meat/day = 1.19 (1.04, 1.37); 50 g processed red meat/day = 1.51 (1.25, 1.83)	Multivariate analyses adjusted for age, ethnicity, smoking, energy intake, alcohol intake, history of HTN and hypercholesterolemia, family history of diabetes, body weight, and physical activity. A diet score was created looking at *trans* fats, glycemic load, cereal fiber, and the ratio of polyunsaturated to saturated fat.

In a 17-year follow-up of 8401 individuals participating in either the Adventist Mortality Study or the Adventist Health Study who were free of diabetes at baseline, those who reported eating meat (defined as red meat, poultry, and fish) at least weekly at the study’s endpoint were 29% more likely to have developed diabetes, compared to those who reported no meat consumption at that time point. Fish intake was uncommon in this cohort and, considered in isolation, was associated with an increase in diabetes risk that did not reach statistical significance (OR = 1.12, 95% CI, 0.88–1.44). Consumption of processed meats (salted fish and frankfurters), adjusted for all other meat consumption, was associated with a 27% increased risk of diabetes, compared with those who avoided processed meats. Those who reported long-term meat consumption (*i.e.*, intake at both the beginning and end of the study period) had a 74% increased risk for developing diabetes, compared with those avoiding meat at both time points. Adjustment for education, physical activity, smoking, and alcohol use did not substantially alter the above findings. Adjustment for body mass index or weight gain attenuated, but did not eliminate, the association between long-term meat consumption and diabetes risk [9].

**Figure 1:**
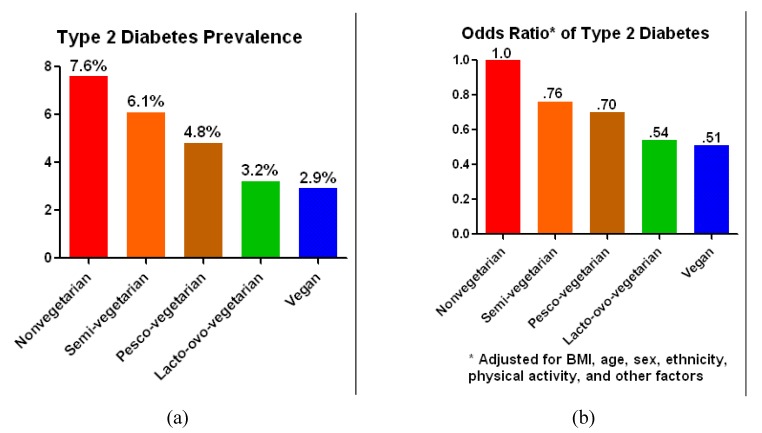
(**a**) Type 2 diabetes prevalence and (**b**) adjusted odds ratio of developing type 2 diabetes among individuals with varying dietary patterns.

The Adventist Health Study-2 included 60,903 Adventists, approximately one-quarter of whom were black; most of the remainder were white. At baseline, diabetes prevalence was 3.2% among individuals consuming no meat, compared with 7.6% for those consuming any sort of meat on a daily basis. Those consuming meat less than weekly and those having no meat other than fish were between these extremes (6.1% and 4.8%, respectively) (Figure 1) [10]. After adjustment for body mass index, physical activity, age, sex, ethnicity, and other factors, the odds ratio of a diagnosis of type 2 diabetes among meat consumers remained approximately twice that of individuals avoiding meat. Those who consumed meat less than once per week or who limited their meat consumption to fish also remained at elevated risk, albeit not so high as for those consuming all types of meat on a daily basis.

A 2-year follow-up period included 41,387 men and women. Compared with those eating meat more than once per week and after adjustment for age, body mass index, gender, ethnicity, income, and education, risk of developing diabetes was significantly lower in vegans (odds ratio = 0.381, 95% CI, 0.236–0.617), lacto-ovo-vegetarians (odds ratio = 0.618, 95% CI, 0.503–0.760), and those consuming red meat or poultry less than once per week (odds ratio = 0.486, 95% CI, 0.312–0.755). Risk was not significantly lower among those who ate fish but no other meats (odds ratio = 0.790, 95% CI, 0.575–1.086) [11]. While the foregoing studies indicate substantially increased risk of diabetes associated with meat consumption independent of body weight, they do not settle the question as to whether this association is mediated by the addition of meat *per se* or by the displacement of plants that may follow the inclusion of meat in the diet.

#### 2.1.1. Risk Associated with Gradations of Meat Consumption

Among meat-consuming populations, the contribution of gradations of meat consumption to diabetes risk has been quantified in several prospective studies, both as an isolated scalar variable and as part of a larger dietary pattern. A 2011 meta-analysis by Pan *et al.* [12], including 442,101 participants and 28,228 diabetes cases, showed that consumption of both unprocessed and processed red meat, as divided into quintiles, was significantly associated with risk of type 2 diabetes. Processed meat was ascertained by questions about use of “bacon”, “hot dogs” and “sausage, salami, bologna, and other processed red meats” on a food frequency questionnaire. The relative risk associated per 100-g serving of unprocessed red meat per day was 1.19 (95% CI, 1.04–1.37). For processed meat, the relative risk associated per 50-gram serving per day was 1.51 (95% CI, 1.25–1.83). The meta-analysis did not consider other types of meat [12]. In population studies that include a sufficient number people who avoid all meats such that comparisons can be made between these people and those who eat red meat, fish, *etc.*, those who avoid all meats have the lowest risks of diabetes. In studies, such as Pan’s [12], that examine gradations of meat intake without a comparison to those who avoid meats altogether, red meat and processed meat stand out as contributors to risk. This study confirmed the results of two prior meta-analyses [13,14].

Among the data sets contributing to this meta-analysis were two cohort studies that separated the risk attributable to meat consumption *per se* from that related to a more complex dietary pattern. In the Nurses’ Health Study I, two major dietary patterns were identified among the 69,554 participants: a “Western” dietary pattern, defined by higher intakes of red and processed meats, sweets, and desserts, french fries, and refined grains, and a “prudent” dietary pattern, characterized by higher intakes of fruits, vegetables, legumes, fish, poultry, and whole grains [1]. After adjustment for age, family history of diabetes, calories, physical activity, body mass index, and other factors, those in the highest quintile of the Western pattern had a 49% (RR = 1.49, 95% CI 1.26–1.76, *P* < 0.001) increased risk of developing diabetes during 14 years of follow-up, compared with those in the lowest quintile [15].

After adjustment for the Western dietary score, the associations between meat intake and diabetes risk remained significant; the relative risk for each added daily meat serving was 1.26 (95% CI, 1.21–1.42) for red meat and 1.38 (95% CI, 1.23–1.56) for processed meat, suggesting, in the study authors’ words, “that these foods are associated with diabetes risk independently of the overall Western pattern”.

In the Nurses’ Health Study II, including 91,246 women followed for eight years, consumption of processed meat five or more times per week was associated with increased risk of type 2 diabetes (RR = 1.91, 95% CI, 1.42–2.57, *P* < 0.001 for trend) [16]. Adjustment for a “Western” dietary pattern (associated with higher intakes of red meat, processed meat, refined grain products, snacks, sweets and desserts, french fries, and pizza) did not materially change this result. For red meat consumption 5 or more times per week, compared with <1 time per week, in a multivariate model with further adjustment for a “Western” dietary pattern, the relative risk of type 2 diabetes was 1.59 (95% CI, 1.01–2.49). These studies indicate that, while a Western dietary pattern is associated with diabetes risk, meat consumption increases diabetes risk independently of dietary pattern.

A separate analysis examined fish consumption among 195,204 adults participating in the Nurses’ Health Study I, the Nurses’ Health Study II, or the Health Professionals Follow-up Study. Those who consumed 5 or more fish servings per week had a 22% increased risk (RR = 1.22, 95% CI, 1.08–1.39, *P* for trend <0.001) for developing diabetes during the 14- to 18-year follow-up period, compared with those who consumed fish less than once per month after adjustment for body mass index, physical activity, family history of diabetes, caloric intake, intakes of saturated and *trans* fats, and other factors [17].

An additional and methodologically distinct study examined diets of participants in the European Prospective Investigation into Cancer and Nutrition Study and the Multiethnic Cohort Study, finding that consumption of fish and meat was higher in individuals with diabetes, compared with those without diabetes [18].

In summary, these studies show that meat consumption is related to diabetes risk. Available data do not exonerate any particular type of meat, and the role of meat consumption in risk appears to be largely independent of its role as part of a dietary pattern.

#### 2.1.2. Mechanisms of Action

Although the value of risk factors does not depend on the identification of mechanisms by which they cause disease (as in the case of increased diabetes risk among certain ethnic groups, for example), the presence of biological mechanisms linking meat consumption with diabetes supports its validity as a *bona fide* risk factor meriting the attention of clinicians and policymakers.

##### 2.1.2.1. Effect on Body Weight

Nearly all observational studies comparing meat-eaters with those who avoid meat show higher body weights among the former group, a finding mirrored in the results of intervention studies using meatless diets [19]. The relatively high fat content and the absence of fiber in meat products typically makes them higher in energy density, compared with most vegetables, fruits, legumes, or grain products [20]. Dietary interventions omitting meat and other animal-derived products typically lead to a reduction in energy intake without increased hunger [20]. The reduction in energy density is not fully compensated for by increased food intake [21,22,23]. Recently published findings from the Chicago Western Electric Study indicated an association between animal protein intake and obesity, suggesting the possibility of mechanisms influencing body weight other than those noted above [24]. The study authors proposed as possible explanatory mechanisms that insulin resistance may be aggravated by the specific amino acids and fat that are particularly abundant in meats and that saturated fatty acids in meat products may increase the insulin response which, in turn, increases the respiratory quotient and reduces fat oxidation.

While low-carbohydrate diets that include meat often cause weight loss, this effect is apparently not due to any special effect of meat consumption, but rather to a reduction in energy intake that comes with the temporary exclusion of broad categories of carbohydrate-containing foods [25].

To the extent that meat’s contribution to overweight and obesity mediates its tendency to increase diabetes risk, adjustment for body weight in studies of diabetes risk is inappropriate and lead to an underestimate of the true degree of risk. However, the tendency of meat consumption to increase body weight can only partially explain the association with diabetes risk, because this association is not fully attenuated after adjustment for body weight [7,9,10,11,12,15,17].

##### 2.1.2.2. Effect on Visceral Fat

Apart from the effect of generalized adiposity, it may be that visceral fat in particular contributes to insulin resistance and risk of type 2 diabetes. A diet eliminating meat was shown to reduce visceral fat and improve insulin sensitivity, compared with a more conventional diabetic diet [26]. A 2006 review article by Hamdy and colleagues suggested that increased visceral adipose tissue is associated with insulin resistance as a result of increased proinflamatory cytokines originated from visceral fat cells and from adipose tissue-resident macrophages [27].

##### 2.1.2.3. Effect on Intracellular Lipid

Studies have suggested that fat accumulation within muscle and liver cells (or the metabolism of these lipids) aggravates insulin resistance which, in turn, contributes to type 2 diabetes [28,29,30]. Meat products are generally fattier than typical grains, legumes, vegetables, and fruits. Nuts and seeds have a high fat content, although lower in saturated fat than most meat products. Not only may dietary fat contribute to intracellular lipid, but high-fat foods also appear to downregulate the genes responsible for mitochondrial oxidative phosphorylation in muscle tissue [31]. Among individuals habitually avoiding animal products, intramyocellular lipid concentrations were significantly lower, compared with age- and weight-matched omnivores (−9.7, 95% CI −16.2 to −3.3, *P* = 0.01) [32]. These studies suggest that the inclusion of meat in the diet contributes a load of dietary fat that enhances intracellular lipid storage and impairs insulin metabolism which increases resistance. Indeed, nondiabetic individuals following an omnivorous diet who then begin a diet omitting animal products have demonstrated increased insulin sensitivity [20], although this may also reflect the effect of weight loss and changes in intake of other food components.

##### 2.1.2.4. Effect on Iron Balance

Meat provides a substantial quantity of heme iron, which is more absorbable, compared with non-heme iron. As a prooxidant, iron encourages the production of reactive oxygen species, which may damage body tissues, including insulin-producing pancreatic cells [33]. Elevated body iron stores are associated with insulin resistance, and even moderately elevated iron stores are associated with increased risk for type 2 diabetes [33,34]. Conversely, reductions in stored iron, through either dietary changes or phlebotomy, increase insulin sensitivity [33,34]. A 2009 review by Liu and colleagues concluded that a reduction in heme iron intake would help prevent insulin resistance, type 2 diabetes, and diabetes complications [35]. In the 2011 meta-analysis cited above [12], intakes of red meat and of heme iron were strongly correlated, and adjustment for heme iron intake attenuated the relationship between red meat and diabetes risk [12].

##### 2.1.2.5. Nitrates in Processed Meats

Processed meats are similar to other meats in their macronutrient content, but may also contain nitrites and sodium, both of which have been advanced as potential explanations for the association between processed meats and diabetes [36].

##### 2.1.2.6. Inflammation

Some have speculated that that apparent deleterious effect of meat consumption on glycemic control and diabetes risk is mediated by inflammation. A 2013 review noted that meat-based diets, or “Western” dietary patterns, were positively associated with biomarkers of inflammation, while fruit- and vegetable-based diets, or “healthy” patterns, were inversely associated with biomarkers of inflammation [37]. Similarly, a 2014 Harvard study reported that as total red meat consumption increased among women from the Nurses’ Health Study, so did biomarkers of inflammation [38].

#### 2.1.3. The Use of Risk Factors in Clinical Practice and Health Policies

Risk factors for diabetes identified in research studies increase vigilance on the part of clinicians, influence recommendations for screening, and encourage at-risk individuals to modify their behavior. Risk factors related to diet can be particularly useful. First, unlike family history, race, and even body weight, they are readily modifiable. Second, they are linked not only to risk of diabetes, but also to risk of cardiovascular disease, which is particularly relevant to diabetes morbidity and mortality.

To put the degree of risk in context, Hispanic ethnicity is associated with not quite twice the risk for developing diabetes, compared with figures for non-Hispanic whites. Data from the National Health Interview Survey (1984–2000) showed that, among males, the lifetime risk at birth for developing diabetes was 45.4% for Hispanics, compared with 26.7% for non-Hispanic whites. Among females, the figures were 52.5% for Hispanics and 31.2% for non-Hispanic whites [39]. Data from cross-sectional and prospective studies suggest that individuals who regularly consume meat products may have up to twice the risk of developing diabetes, compared with individuals who avoid meat entirely [7,8,9,10,11].

Individuals who eat meat regularly also tend to have higher plasma total and low density lipoprotein cholesterol concentrations [40], higher blood pressure values [41], higher risk of hypertension [41], and higher body weight [19], all of which contribute to cardiovascular risk, the principal danger in diabetes. All of these conditions improve when meat is no longer consumed [20,42,43,44]. That is, lipid concentrations improve [40], due to the reduction of saturated fatty acid and cholesterol intake and the increase in soluble fiber and other plant constituents shown to alter plasma lipids; blood pressure falls [41], due to the addition of components of plant foods (e.g., potassium) and the changing macronutrient content of the diet as evidenced by the Dietary Approaches to Stop Hypertension (DASH) study, which was in part inspired by observations of lower blood pressure among vegetarians [45]; and body weight diminishes [19,44,46], due to reduced energy density of the diet and, to a lesser extent, increased post-prandial thermogenesis [20].

As we have noted, the apparent problems of meat consumption are not limited to red meat. Although the saturated fat load in poultry and fish products may be lower than in typical red meats and omega-3 fatty acids account for a portion of the fat in many fish species, saturated fat content is nonetheless generally higher in poultry and fish than in typical vegetables, fruits, legumes, and grains. Poultry and fish provide no fiber or complex carbohydrate and tend to displace foods that would provide these nutrients, which may account for the differences in body weight and in diabetes prevalence noted between fish-eaters and those who avoid all meats [10]. Per capita red meat consumption has fallen in the U.S. in recent decades, while a compensatory increase in poultry and fish consumption has led to overall increases in total per capita meat consumption [47].

Meat consumption as a categorical variable is easily ascertainable by self-report. As such it is clinically useful as a diabetes risk factor. While it is theoretically possible to use dietary patterns or gradations of meat intake as risk factors, the detailed dietary assessments that may be required to determine the degree of risk are cumbersome in practice. Nonetheless, the fact that studies of gradations of meat consumption indicate a dose-response relationship with diabetes risk supports the validity of the use of meat consumption as a risk factor.

Once identified, at-risk individuals can be encouraged to familiarize themselves with meatless options through recipes, cookbooks, online resources, and classes, and their medical care-givers can enlist the expertise of dietetic professionals in ensuring complete nutrition and providing group or individual instruction on menu planning and related topics. In the research setting, plant-based dietetic group instruction and support have helped at-risk individuals lose weight over both the short and long term and improve insulin sensitivity [20,46].

Strengths of the body of data contributing to this analysis include large sample sizes and strong methodology in prospective studies, consistent findings among studies, and a dose-response relationship. Limitations include the fact that two of the cited Adventist cohorts were restricted to white participants. However, confidence in the generalizability of these findings comes from consistency with findings in non-Adventist populations. It is possible those who abstain from meat also make other lifestyle choices that benefit health and may in turn confound the findings of the reduced risk for type 2 diabetes. However, the included studies have accounted for potential confounding factors to a greater or lesser degree. Some reassurance derives from the use of stricter controls in the more recent studies, which adjusted for age, smoking, calorie and alcohol intake, and exercise, as well as in the similarity of results found in clinical trials, including better glycemic control and reduced body weight. While one may be tempted to combine data from the published studies in a meta-analysis, the data sets and statistical procedures vary to the point that such an analysis would be based on so many assumptions that it would not enhance our understanding of the relationships under study.

## 3. Conclusions

Meat consumption is consistently associated with diabetes risk. Dietary habits are readily modifiable, but individuals and clinicians will consider dietary changes only if they are aware of the potential benefits of doing so. The foregoing review indicates that the identification of meat consumption as a risk factor for diabetes provides helpful guidance for clinicians and at-risk individuals, and sets the stage for beneficial behavioral changes.
