# Correction: Barnard, N., *et al*. Meat Consumption as a Risk Factor for Type 2 Diabetes. *Nutrients* 2014, *6*, 897-910

**DOI:** 10.3390/nu6031181

**Published:** 2014-03-21

**Authors:** Neal Barnard, Susan Levin, Caroline Trapp

**Affiliations:** 1 Physicians Committee for Responsible Medicine, The George Washington University School of Medicine, 5100 Wisconsin Ave NW, Washington, DC 20016, USA; E-Mail: nbarnard@pcrm.org; 2 Nutrition Education, Physicians Committee for Responsible Medicine, 5100 Wisconsin Ave NW, Washington, DC 20016, USA; E-Mail: ctrapp@pcrm.org

We have found two inadvertent errors in our paper published in Nutrients [1].

On page 909, reference number 38, the first author should read “Ley” and not “Lev”. Additionally, the year cited should read “2014” and not “2010”. The final reference should read: 

38. Ley, S.H.; Sun, Q.; Willett, W.C.; Eliassen, A.H.; Wu, K.; Pan, A.; Grodstein, F.; Hu, F.B. Associations between red meat intake and biomarkers of inflammation and glucose metabolism in women. *Am. J. Clin. Nutr*. **2014**, *99*, 352–360.

These changes have no material impact on the conclusions of our paper. We apologize to our readers.

## References

[1] Barnard N., Levin S., Trapp C. (2014). Meat Consumption as a Risk Factor for Type 2 Diabetes. Nutrients.

