# Correction: Barnard, N., *et al*. Meat Consumption as a Risk Factor for Type 2 Diabetes. *Nutrients* 2014, *6*, 897–910

**DOI:** 10.3390/nu6104317

**Published:** 2014-10-16

**Authors:** Neal Barnard, Susan Levin, Caroline Trapp

**Affiliations:** 1 Physicians Committee for Responsible Medicine, The George Washington University School of Medicine, 5100 Wisconsin Ave NW, Washington, DC 20016, USA; E-Mail: nbarnard@pcrm.org; 2 Nutrition Education, Physicians Committee for Responsible Medicine, 5100 Wisconsin Ave NW, Washington, DC 20016, USA; E-Mail: ctrapp@pcrm.org

We have found some inadvertent errors in our paper published in Nutrients [1]. This is a second published correction, the first correction can be found [2].

On page 900, the table heading should read “*Risk for diabetes in meat-eaters, compared with non-meat-eaters*” and not “*Meat as a Categorical Variable*”.

On page 900, the study titled “*Adventist Mortality Study and Adventist Health Study-1 Tonstad et al. (2013) [11]*” requires a change in the citation to “*9*”. The full text should read, “*Adventist Mortality Study and Adventist Health Study-1 Vang et al. (2008) [9]*”.

On page 900, the study titled “*Adventist Health Study-2 Tonstad et al. (2009) [10]*” requires a change in the Findings column. The text should read “*Odds ratio and 95% CI for diabetes diagnosis: 1.85 (1.67, 2.04)*” and not “*Odds ratio and 95% CI for diabetes diagnosis: 0.54 (0.49, 0.60)*”.

On page 900, the study titled “*Adventist Health Study-2 Tonstad et al. (2013) [11]*” requires a change in the Findings column. The text should read “*Odds ratio with 95% CI for diabetes diagnosis: 1.62 (1.32, 1.99)*” and not “*Odds ratio with 95% CI for diabetes diagnosis: 0.618 (0.0503, 0.760)*”.

On page 900, the study titled “*Meta-analysis Pan et al. (2011) [12]*” should read “*Pan et al. (2011) [12]*”. The “*+ D1*” in the Findings column of this study should be deleted. The text should read “*Relative ratios and 95% CI for diabetes diagnosis*”. Additionally, we would like to insert a table heading before this study. The text should read “*Meta-analysis of risk of developing diabetes related to daily meat servings*”.

The fully corrected table should appear as follows:

**Table 1:** Published studies of the relationship between meat consumption and risk of type 2 diabetes.

Risk for Diabetes in Meat-Eaters, Compared with Non-Meat-Eaters				
Study	Observation Period	Population	Findings	Adjustments
Adventist Mortality Study Snowdon *et al.* (1985) [7]	1960	24,673 white Seventh-day Adventists	Prevalence ratio and 95% CI for diabetes diagnosis: Men = 1.8 (1.3, 2.5); Women = 1.4 (1.2, 1.8)	Age and body weight
Adventist Mortality Study Snowdon *et al.* (1985) [7]	21-year follow-up	24,673 white Seventh-day Adventists	Relative risk for diabetes on death certificate: Men = 2.2 (1.5, 3.4); Women = 1.4 (1.0, 1.9)	Age
Adventist Health Study-1 Fraser (1999) [8]	1976	34,192 Seventh-day Adventists in California	Odds ratio and 95% CI for diabetes diagnosis: Men = 1.97 (1.56, 2.47, *p* = 0.0001); Women = 1.93 (1.65, 2.25, *p* = 0.0001)	Age
Adventist Mortality Study and Adventist Health Study-1 Vang *et al.* (2013) [9]	17-year follow-up	8401 Seventh-day Adventists	Odds ratio with 95% CI for diabetes diagnosis: 1.29 (1.08, 1.55)	Age and gender
Adventist Health Study-2 Tonstad *et al.* (2009) [10]	2002–2006	60,903 Seventh-day Adventists in North America	Odds ratio and 95% CI for diabetes diagnosis: 1.85 (1.67, 2.04)	Age, sex, ethnicity, education, income, physical activity, television watching, sleep habits, alcohol use, and body mass index
Adventist Health Study-2 Tonstad *et al.* (2013) [11]	2-year follow-up	41,387 Seventh-day Adventists	Odds ratio with 95% CI for diabetes diagnosis: 1.62 (1.32, 1.99)	Age, body mass index, gender, ethnicity, income, and education
**Meta-Analysis of Risk of Developing Diabetes Related to Daily Meat Servings**				
Pan *et al.* (2011) [12]	4.6 to 28 years follow-up	442,101	Relative ratios and 95% CI for diabetes diagnosis: 100 g unprocessed red meat/day = 1.19 (1.04, 1.37); 50 g processed red meat/day = 1.51 (1.25, 1.83)	Multivariate analyses adjusted for age, ethnicity, smoking, energy intake, alcohol intake, history of HTN and hypercholesterolemia, family history of diabetes, body weight, and physical activity. A diet score was created looking at *trans* fats, glycemic load, cereal fiber, and the ratio of polyunsaturated to saturated fat.

On page 902, the citation that reads “*[1]*” should read “*[15]*”. The full, corrected text should read “*In the Nurses’ Health Study I, two major dietary patterns were identified among the 69,544 participants: a “Western” dietary pattern, defined by higher intakes of red and processed meats, sweets, and desserts, French fries, and refined grains, and a “prudent” dietary pattern, characterized by higher intakes of fruits, vegetables, legumes, fish, poultry, and whole grains [15]*”.

These changes have no material impact on the conclusions of our paper. We apologize to our readers.
